# Computed tomography of the shoulders in patients with obstetric brachial plexus injuries: a retrospective study

**DOI:** 10.1186/1750-1164-2-4

**Published:** 2008-11-07

**Authors:** Rahul K Nath, Andrea D Humphries

**Affiliations:** 1 Texas Nerve and Paralysis Institute, 6400 Fannin Street, Suite 2420, Houston, TX 77030, USA

## Abstract

**Background:**

Scapular hypoplasia, elevation, and rotation (SHEAR) deformity and posterior subluxation of the humeral head are common tertiary sequelae of obstetric brachial plexus injuries (OBPI). Interpretations of images from bilateral computed tomography (CT) scans of the upper extremities are critical to the diagnosis and treatment plan for patients with these bony deformities resulting from OBPI.

**Methods:**

We conducted a retrospective study to investigate the accuracy of radiologic reports in the diagnosis of SHEAR or posterior subluxation of the humeral head in OBPI patients. CT studies from 43 consecutive patients over a 33-month period were used in the study. For each patient, we compared the results from the radiologic report to those from a clinical examination given by the attending surgeon and to measurements taken from the CT studies by biomedical researchers.

**Results:**

A comparison of SHEAR measured from the 3-D CT images to the diagnoses from the radiologists, revealed that only 40% of the radiological reports were accurate. However, there was a direct correlation between the use of the 3-D CT images and an accurate SHEAR diagnosis by the radiologists (p < 0.0001). When posterior subluxation was measured in the affected and contralateral shoulders, 93% of the patients that had greater than a 10% difference between the two shoulders did not have their deformity diagnosed. The radiological reports diagnosed 17% of these patients with a 'normal' shoulder. Only 5% of the reports were complete, accurately diagnosing SHEAR in addition to posterior subluxation.

**Conclusion:**

Due to the low incidence rate of OBPI, many radiologists may be unfamiliar with the sequelae of these injuries. It is therefore critical that radiologists are made aware of the importance of an accurate measurement and diagnosis of the SHEAR deformity. Due to their lack of completeness, the radiological reports in this study did not significantly contribute to the clinical care of the patients. In order for OBPI patients to receive the highest standard of care, the final diagnosis from their radiological imaging should be deferred to a brachial plexus specialist who is experienced with these types of injuries.

## Background

The upper nerve roots of the brachial plexus are commonly injured in patients with obstetric brachial plexus injuries. This results in the diminished development of external rotators and abductors which no longer counteract the opposing muscle groups. In the developing shoulder girdle, these muscular imbalances lead to glenohumeral dysplasia and instability [1]. A medial rotation contracture develops due to the unopposed contraction of the internal rotators and often progresses to a fixed medial rotation position of the humerus and posterior subluxation of the humeral head [1,2]. Irregularities of the scapula in these patients are also very common [3-5]. Recently, we have described in detail the characteristic scapular hypoplasia, elevation, and rotation (SHEAR) deformity that is a common tertiary sequela of obstetric brachial plexus lesions [4]. Briefly, the abnormal lateral and anterior rotation of the scapula, that is a result of the weakened scapular stabilizing muscles, deforms the clavicle and acromioclavicular triangle (see reference 3 for a more complete description). The scapular elevation rotates the superior surface of the distal third of the clavicle anteriorly, and the lateral scapular migration bends the entire distal third of the clavicle forward. As a result, the distal acromioclavicular triangle is tilted and assumes a more superior and anterior placement.

To measure all the aspects of the scapular deformity, three-dimensional reconstructed CT images are used as previously described [4,6]. From the anterior/posterior view of the 3-D reconstructed CT images, the percent of the total area of the scapula, superior border and medial border that is visible above the clavicle is measured [4]. Additionally, the width of the hypoplastic scapula compared to the contralateral scapula is measured from the posterior scapular views. Finally, the angles of superior displacement, downward/upward rotation, and anterior/posterior rotation are also calculated from posterior view and superior outlet images [4].

**Figure 1:**
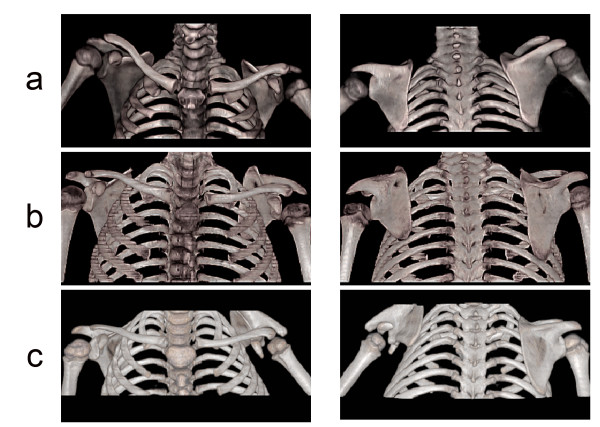
**Three-dimensional CT Reconstructions Demonstrating Progressive SHEAR Deformities Misdiagnosed as Normal**. Anterior (left) and posterior (right) three-dimensional CT reconstructions of bilateral CT scans of the upper extremities are depicted. Grade 2 (a), grade 3 (b), and grade 4 (c) SHEAR deformities are present in three OBPI patients that have 9.6%, 27.2% and 56.5% of the scapular body visible over the clavicle compared to the contralateral shoulder, respectively.

In our previous study, we found that the percentage of the total area of the affected scapula visible over the clavicle correlated with most of the parameters discussed above, and was a good indication of a patient's grade of SHEAR [4]. Therefore, the different stages of SHEAR (Grade 0–4) can be assessed using the anterior view of the bilateral 3-D CT radiographs. When compared to the contralateral shoulder, a shoulder with grade 0 SHEAR has less than 2% of the total scapular area visible above the clavicle, with grade 1 SHEAR, 2–3.6% of the scapula is visible, with grade 2 SHEAR, 3.6–20% is visible, with grade 3 SHEAR, 20–45% is visible and with grade 4 SHEAR, more than 45% of the scapula is visible above the clavicle. Figure 1 shows examples of grades 2, 3, and 4 SHEAR deformity seen in this study. In addition, our recent findings that SHEAR strongly correlates with humeral head posterior subluxation provides a convenient diagnostic tool that will allow for a rational treatment plan to be developed [6].

Clinical evaluations of OBPI patients are often corroborated by radiographic imaging techniques. Consequently, interpretation and quantification of patient CT scans contribute to the diagnosis and treatment plan, and it is therefore an essential component in managing care of the patient. Several other groups have evaluated the benefit of dual interpretations of radiological results [7-13]. In one group of studies, a dual interpretation of the radiological findings were repeatedly found to have no impact on patient care as there were no significant differences between the attending physician's interpretation of these images compared with that of the radiologist [10-13]. Routine radiological consultations were deemed extraneous and unnecessary. Another group of studies noted the consistently inaccurate nature of the radiological findings compared to that of the attending physician [7-9]. Interestingly, two of these reports also pointed out that interpretations of musculoskeletal radiographs can be particularly problematic [8,11].

The purpose of this study was to report the accuracy and completeness of 43 radiological reports from patients with obstetric brachial plexus injuries. Our results establish a basis to question the ability of these reports to provide the critical information needed to make decisions regarding clinical treatment.

## Methods

We conducted a retrospective study on the accuracy and completeness of computerized tomography (CT) scans from 43 OBPI patients. Patients with a grade 1 to 4 SHEAR deformity seen between the months of March 2004 to December 2006, that had no record of previous bony surgeries prior to the date of the CT, were included in the study. Patients were excluded if their complete CT records were not available for review. Twenty-eight patients were female and fifteen were male with a mean age of 5.4 years (range 1.0 – 12.5). Thirty-one patients had obstetric brachial plexus injuries on the right side and 12 patients had injuries on the left side. The lead author, who is a surgeon with 10 years of experience with over 4,000 OBPI patients, performed all clinical evaluations. Upon initial clinical evaluation, all 43 patients presented with a medial rotation contracture of the shoulder, palpable posterior subluxation of the humeral head, and SHEAR deformities ranging from Grades 1–4.

Prescriptions for bilateral CT scans, without contrast, of the upper extremities and three-dimensional surface rendering with bone algorithm were issued for each patient to confirm clinical evaluations and to aid in the planning of appropriate surgical procedures. Instructions on using the 3-D rendering to identify and classify the SHEAR deformity common to obstetric brachial plexus injuries accompanied all CT prescriptions.

SHEAR and posterior subluxation of the affected and contralateral shoulders were measured from the CT studies independently of the brachial plexus specialist and the radiologists. From the anterior view, the percent of the total area of the scapula visible above the clavicle in the affected shoulder compared to the contralateral shoulder was measured using graphic software (Universal Desktop Ruler, AVP-Soft.com) as described previously [4]. From the axial view, humeral head posterior subluxation was measured as previously described [14]. Briefly, the maximum diameter of the humeral head was measured, as was the distance from the bisecting scapular line to the anterior aspect of the humeral head. Posterior subluxation was calculated from the ratio of the distance from the scapular line to the anterior aspect of the humeral head divided by the humeral head diameter multiplied by 100. The difference between the contralateral and affected shoulders is reported here as the difference in posterior subluxation or the difference in the percent of the humeral head anterior to the scapular line (PHHA) [14,15]. Only shoulders with a greater than 10% difference between the affected versus contralateral shoulders were evaluated for accuracy in the radiological reports.

Reports from the radiologist as well as the clinical diagnosis by the attending physician (RKN) were compared directly to the SHEAR and posterior subluxation shoulder measurements. An accurate diagnosis (within one grade of SHEAR) was scored with a "1", and a "0" denoted an omission or an inaccurate diagnosis [see Additional file 1]. In addition, the radiological reports were scored based on the following parameters: (1) reference to the 3-D CT reconstructions in the report, (2) the detection of glenohumeral joint subluxation when > 10% difference in PHHA was present in the affected compared to the contralateral shoulder, (3) if the affected shoulder was referred to as 'normal' or 'without abnormality' (excluding all patients with ≤ 10% difference in PHHA in the affected shoulder compared to the normal one), and (4) if the report accurately diagnosed SHEAR and PHHA and was considered to be complete.

Kappa statistics with a 95% confidence interval were calculated to quantify interrater agreement [16]. Spearman's correlation tests were conducted to determine if there was a significant association between defined categorical variables. A two-tailed p-value < 0.05 was considered significant. A significant difference in the mean percentage of the humeral head anterior to the scapular line and percent SHEAR in affected versus normal shoulders were calculated using the paired Student's t-test, and a one-tailed p-value < 0.05 was considered significant. The above calculations were performed using Micrsoft Excel plus Analyse-It (Analyse-It Software, Ltd., Leeds, England).

## Results

### Interrater agreement

The radiological reports from forty-three bilateral CT scans with three-dimensional renderings of patients with obstetric brachial plexus injuries were analyzed. During the 33 month period of this study, we consistently received reports from different radiologists that failed to diagnose the bony deformities in our OBPI patients. SHEAR and posterior subluxation of the humeral head were often misdiagnosed or completely overlooked in the radiological reports. Compared to the SHEAR measurements taken from the 3-D CT images, there was no interrater agreement between the accuracy of the radiological reports and the clinical diagnosis of SHEAR from the surgeon (kappa = 0.03). In addition, 17% of the radiological reports indicated that the damaged shoulder girdle appeared 'normal' or there were 'no detectable abnormalities.' This excluded the cases where a patient had less than a 10% difference in measured posterior subluxation between the two shoulders. Only 5% of the radiological reports were complete with an accurate diagnosis of SHEAR and posterior subluxation [see Additional file 1]. Likewise, there was no association between a 'normal' reading by the radiologist and the use of the 3-D CT images (p = 0.31). This indicates that a misdiagnosis of 'normal' was less likely to occur when the radiologist utilized the 3-D images.

### Diagnosis of SHEAR

The average SHEAR in the affected shoulders from the 43 OBPI patients, measured by percent scapular elevation, was 16.4% (range 2.1 to 56.5) and differed significantly from the measurements taken in the contralateral shoulder (p < 0.0001). In agreement with our previous findings, there was no correlation between the age of the patients and the degree of SHEAR [4]. Only 17 of the 43 formal reports diagnosed SHEAR accurately (40%) compared to the 98% accuracy of the clinical diagnosis from the attending physician. To determine the reason for the low accuracy of the radiological diagnosis of SHEAR, we looked at how effectively the radiologists were using the 3-D reconstructed images. A relatively high proportion of radiologists (77%) referred to the three-dimensional reconstruction of the upper extremities in their evaluations, and the use of the 3-D CT images was associated with an accurate diagnosis of SHEAR (p < 0.001). There was not a single case where SHEAR was diagnosed without the use of the 3-D CT reconstructions.

We grouped the patients according to their measured grade of SHEAR and calculated the accuracy rates with 95% confidence intervals from the radiologists and the attending physician (Table 1). The brachial plexus specialist consistently had a higher accuracy rate, which reached 100% accuracy for patients with SHEAR grades 2–4 (Table 1). Only in the higher grades of SHEAR (3 and 4) did the radiologists have a 50% accuracy rate or higher.

**Table 1:** Percent accuracy of radiological and clinical assessments for different grades of SHEAR compared to measurements taken from individual CT studies.

	**Radiological Reports**		**Initial Diagnosis by Specialist**	
	
**SHEAR GRADE**	**% Accuracy**	**95% Confidence Interval**	**% Accuracy**	**95% Confidence Interval**
1	0		80	(44.9 to 115.1)
2	36	(17.2 to 54.8)	100	
3	66.7	(35.1 to 96.9)	100	
4	50	(1 to 99)	100	

Three cases, patients 2, 24 and 39, where severe SHEAR was present and was not mentioned in the radiological reports are depicted in Figure 1. The anterior and posterior 3-D reconstructions with measured SHEAR grades of 2, 3 and 4 are shown (Figure 1a–c, respectively). The radiological assessment of the patient in Figure 1a noted a 'negative CT of the bilateral shoulders.' This is consistent with our findings that the diagnosis of SHEAR grades 1 and 2 had lower accuracy rates in the radiological reports (Table 1). However, the radiological assessment of the patients in Figure 1b and 1c also did not mention the SHEAR deformity or attempt to measure the percent of the scapula visible above the clavicle in the affected shoulder even though the 3-D reconstructions are mentioned in the radiological reports.

### Diagnosis of posterior subluxation of the humeral head

**Figure 2:**
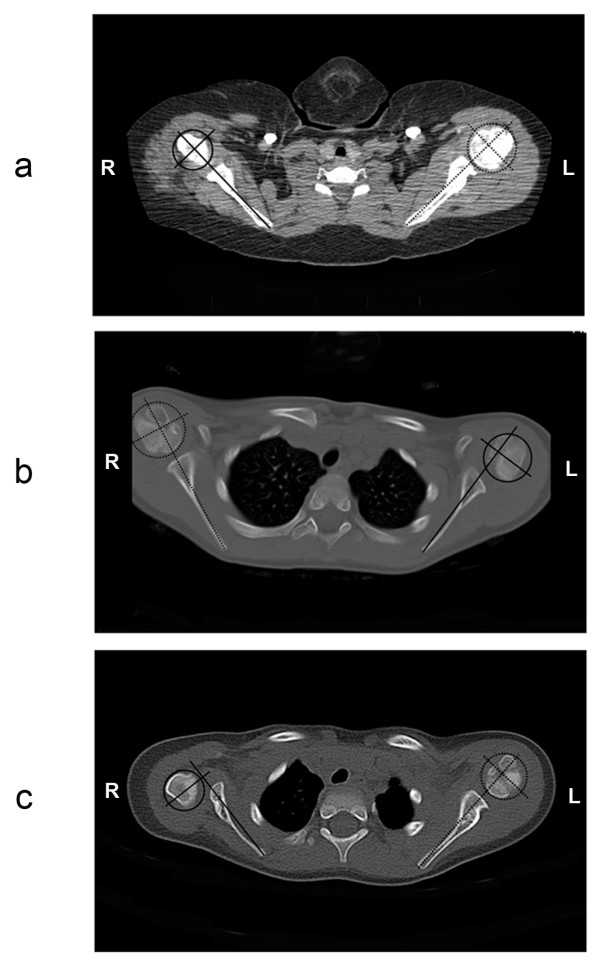
**CT Scans of Three Patients with Severe Posterior Subluxation of the Glenohumeral Joint**. Bilateral axial CT images of the glenohumeral joints from three OBPI patients with increasing severity of posterior subluxation of the affected humeral head are shown. The difference in the percentage of humeral head posterior subluxation in the affected shoulder (solid lines) compared to the normal shoulder (dashed lines) was measured as 3.9% (a), 15.7% (b), and 53% (c) using the indicated lines as described previously [14].

Clinical evaluations revealed palpable posterior subluxation in the glenohumeral joints of all 43 OBPI patients. The mean difference in posterior subluxation in the affected shoulder compared to the normal shoulder was 20% (range 0.4 to 60.9%). The mean posterior subluxation was significantly higher in the affected shoulder compared to the contralateral shoulder (p < 0.0001). Thirteen of the affected shoulders had less than a 10% difference in posterior subluxation compared to the normal shoulder. 93% of the remaining 30 shoulders were not diagnosed with posterior subluxation. It should be noted that 10 out of the 43 OPBI patients in the study were under the age of three years. CT images could underscore the cartilage that would be present in the shoulders of these patients and may not accurately reflect the level of posterior subluxation. Figure 2 demonstrates the progression of severity in posterior subluxation that was overlooked in radiological reports from patients 2, 3 and 24. Measurements taken from axial CT images in the normal shoulder (dashed lines) and the affected shoulder (solid lines) showed there was a 3.9%, 15.7% and 53% difference in posterior subluxation in the affected shoulder compared to the contralateral shoulder (Figure 2a–c, respectively). The reports from the patients in Figure 2b and 2c, respectively, inaccurately concluded that there was 'no evidence of subluxation', and the ' [affected] humeral head appears seated.'

## Discussion

Many radiologists are unfamiliar with the bony sequelae of obstetric injury to the brachial plexus. The CT is a necessary tool to diagnose the severity of the bony deformities, such as SHEAR, commonly seen in OBPI patients. With instruction, the quantification of SHEAR from the three-dimensional reconstruction of CT scans is relatively straightforward. For example, prior to reviewing the 3-D images, one radiologist reported a 'normal CT scan of the left and right shoulders.' However, after examining the 3-D reconstruction as we requested, the radiologist added an addendum to his final report that correctly measured and diagnosed a Grade 2 SHEAR deformity in the same patient. We also found that the lower grades of SHEAR were particularly challenging to diagnose correctly. Accuracy rates in the radiological reports were at or above 50% only when grades 3 and 4 scapular deformities were present (Table 1). The grading system for the SHEAR deformity aids in the diagnosis and evaluation of the bony sequelae in OBPI patients. As the scapula rotates, the position of the acromio-clavicular joint changes and eventually results in the impingment of the humeral head by the distal acromion and clavicle. The anatomical changes in the shoulder that are associated with the SHEAR deformity not only limit range of motion, but also contribute to the medial rotation contractures that are often seen in OBPI patients [17]. We have shown that OPBI patients who underwent a novel surgical procedure called the triangle tilt to correct the SHEAR deformity had improvements in the posture of the affected arm at rest and active shoulder function one year post-surgery [18]. We also observed improvements in shoulder anatomy in post-operative CT exams [18].

Posterior subluxation of the humeral head in relation to the glenoid fossa is another common tertiary deformity of obstetric brachial plexus injuries [19]. We have found that a diagnosis of SHEAR has a strong correlation with the presence of posterior subluxation [4]. Yet, even in cases where the humeral head was completely dislocated from the glenohumeral joint, the patients were described as having normal shoulders in their CT reports (Figure 2). The etiology of this problem may relate to the recumbent position that is required for CT imaging. In this position, inferior and posterior subluxation and dislocation may be convoluted. A recent review on the use of recumbent imaging for evaluating shoulder disorders found that an internally rotated shoulder, the anatomical basis for the humeral head subluxation, can distort the imaging outcome and be a source of diagnostic error for the radiologist or clinician analyzing the shoulder pathology [20].

A common theme seen in the CT reports of these patients is a failure to recognize even the most severe bony sequelae of an obstetric brachial plexus injury. OBPI lesions occur at a rate of 0.4 to 2.5 per 1000 live births, and 80% of these injuries resolve without long-term effects to the injured shoulder girdle [21]. The low incidence of this condition may contribute to the lack of knowledge about its clinical implications. While the brachial plexus specialist is ultimately responsible for the assessment of the radiological studies of each patient, a radiological report that is inaccurate, incomplete, or does not defer the final diagnosis to the specialist can actually hinder the course of treatment for a child when their deformities demand surgical intervention. These two quick measurements of scapular elevation and posterior subluxation are vital to the confirmation of the clinical diagnosis and in the pre-surgical planning stages. The high percent accuracy of the clinical diagnosis compared to the measured grades of SHEAR, and the 60% inaccuracy rates of the radiological reports, lead us to conclude that the accuracy and completeness of the reports were inadequate in their ability to provide the necessary information to contribute to decisions regarding clinical treatment.

## Conclusion

Due to the low incidence of obstetric brachial plexus injuries (0.4 to 2.5 per 1000 live births, [21]), many radiologists may not be familiar with the bony sequelae that frequently result from these types of injuries. The severity of these bony deformities worsens with time causing loss in shoulder function and in the quality of life. Patients with untreated OBPI complications report severely impaired shoulder function due to pain. Inaccurate interpretations of CT scans can postpone treatment and prolong patient disabilities or suffering. Accurate interpretations of the CT scans from OBPI patients are critical in the diagnoses of these deformities. We found that only a small percentage of the radiological reports for the 43 OBPI patients in our study gave an accurate diagnosis for posterior subluxation of the humeral head and SHEAR. Therefore, we recommend that a specialist in brachial plexus injuries review CT scans from OPBI patients to ensure the highest level of care.

## Competing interests

The authors declare that they have no competing interests.

## Authors' contributions

RKN conceived of the study, participated in its design and coordination, performed the clinical evaluations, and edited the manuscript. ADH participated in the design of the study, collected pertinent data, performed all measurements, performed the statistical analysis, and drafted the manuscript. All authors read and approved the final manuscript.

## Supplementary Material

Additional file 1 Comparison of 43 formal computed tomography reports and clinical evaluations with radiological measurements. Data obtained from 43 formal computed tomography reports, compared with clinical evaluations and radiological measurements. Click here for file
