# Supplementary material for: Computed tomography of the shoulders in patients with obstetric brachial plexus injuries: a retrospective study
Source: Ann Surg Innov Res. 2008 Nov 7;2:4. doi: 10.1186/1750-1164-2-4 (PMC2588615; doi:10.1186/1750-1164-2-4)
Supplement: Additional file 1 — Comparison of 43 formal computed tomography reports and clinical evaluations with radiological measurements. Data obtained from 43 formal computed tomography reports, compared with clinical evaluations and radiological measurements. [file 1750-1164-2-4-S1.doc]

Supplemental File 1. Comparison of 43 formal computed tomography reports and clinical evaluations with radiological measurements.

| |  | | | **Accuracy of Radiological Reports** | | | | | | | | **Shoulder Measurements** | | | | | --- | --- | --- | --- | --- | --- | --- | --- | --- | --- | --- | --- | --- | --- | --- | | **Patient** | **Side** | **Age (yrs.)** | **Clinical Diagnosis of SHEAR** | **Radiological Diagnosis of SHEAR^a^** | **Accuracy of Clinical Diagnosis of SHEAR ^a^** | **Accuracy of Radiological SHEAR Diagnsosis ^a^** | **Reference to 3D CT** | **Radiological Diagnosis of PHHA^b^** | **Normal Reading by Radiologist** | **Complete Radiological Report** |  | **PHHA^c^** | **SHEAR^d^** | **SHEAR Grade** | | 1 | R | 7.6 | 4 | n/a | 0 | 0 | 0 | 0 | 0 | 0 |  | **28.6** | **2.7** | 1 | | 2 | L | 2.9 | 4 | n/a | 1 | 0 | 1 | 0 | 0 | 0 | 40.2 | 56.5 | 4 | | 3 | R | 2.4 | 2 | n/a | 1 | 0 | 1 | 0 | 0 | 0 | 53.0 | 7.1 | 2 | | 4 | L | 7.9 | 1 | 2 | 1 | 1 | 1 | 0 | 0 | 0 | 14.6 | 12.2 | 2 | | 5 | R | 4.3 | 2 | n/a | 1 | 0 | 0 | 0 | 1 | 0 | 14.0 | 6.9 | 2 | | 6 | R | 6.9 | 2 | n/a | 1 | 0 | 1 | n/a | 0 | 0 | 0.4 | 6.8 | 2 | | 7 | R | 7.5 | 3 | 2 | 1 | 1 | 1 | 0 | 0 | 0 | 20.2 | 4.3 | 2 | | 8 | R | 1.3 | 2 | n/a | 1 | 0 | 1 | 0 | 1 | 0 | 22.5 | 5.7 | 2 | | 9 | L | 3.2 | 3 | n/a | 1 | 0 | 1 | n/a | 0 | 0 | 5.8 | 6.5 | 2 | | 10 | L | 6.1 | 3 | 3 | 1 | 1 | 1 | n/a | 0 | 0 | 4.6 | 23.7 | 3 | | 11 | L | 1.7 | 2 | n/a | 1 | 0 | 1 | 0 | 0 | 0 | 12.0 | 2.1 | 1 | | 12 | R | 10.8 | 3 | 3 | 1 | 1 | 1 | 0 | 0 | 0 | 11.6 | 38.3 | 3 | | 13 | R | 7.7 | 2 | n/a | 1 | 0 | 1 | n/a | 0 | 0 | 3.9 | 2.3 | 1 | | 14 | L | 5.8 | 3 | n/a | 1 | 0 | 0 | 0 | 1 | 0 | 14.2 | 9.6 | 2 | | 15 | R | 7.1 | 3 | n/a | 1 | 0 | 1 | 0 | 1 | 0 | 27.8 | 20.2 | 3 | | 16 | R | 5.5 | 3 | 3 | 1 | 1 | 1 | n/a | 0 | 0 | 9.2 | 22.9 | 3 | | 17 | R | 6.8 | 3 | 3 | 1 | 1 | 1 | 0 | 0 | 0 | 27.0 | 51.9 | 4 | | 18 | R | 1.0 | 2 | n/a | 1 | 0 | 0 | 0 | 0 | 0 | 22.0 | 18.7 | 2 | | 19 | L | 4.0 | 3 | 3 | 1 | 1 | 1 | 0 | 0 | 0 | 36.3 | 37.5 | 3 | | 20 | R | 2.0 | 3 | n/a | 1 | 0 | 1 | n/a | 0 | 0 | 2.9 | 3.9 | 2 | | 21 | R | 7.3 | 2 | n/a | 1 | 0 | 1 | 0 | 1 | 0 | 38.5 | 14.5 | 2 | | 22 | L | 7.3 | 3 | 3 | 1 | 1 | 1 | 0 | 0 | 0 | 15.7 | 25.3 | 3 | | 23 | R | 7.9 | 1 | n/a | 1 | 0 | 0 | 0 | 0 | 0 | 17.8 | 2.9 | 1 | | 24 | R | 10.7 | 2 | n/a | 1 | 0 | 1 | 0 | 0 | 0 | 15.2 | 6.0 | 2 | | 25 | R | 2.8 | 2 | 2 | 1 | 1 | 1 | 0 | 0 | 0 | 10.8 | 9.7 | 2 | | 26 | R | 1.1 | 3 | 2 | 1 | 1 | 1 | 0 | 0 | 0 | 26.7 | 19.9 | 2 | | 27 | R | 10.7 | 2 | n/a | 1 | 0 | 0 | 0 | 0 | 0 | 10.1 | 7.2 | 2 | | 28 | R | 7.0 | 2 | n/a | 1 | 0 | 1 | 0 | 0 | 0 |  | 19.4 | 10.9 | 2 | | 29 | R | 1.8 | 2 | 2 | 1 | 1 | 1 | 0 | 0 | 0 | 16.1 | 15.6 | 2 | | 30 | R | 1.0 | 1 | n/a | 1 | 0 | 0 | n/a | 0 | 0 | 2.1 | 6.2 | 2 | | 31 | L | 4.0 | 3 | 2 | 1 | 1 | 1 | n/a | 0 | 0 | 5.0 | 4.6 | 2 | | 32 | R | 4.4 | 2 | n/a | 1 | 0 | 0 | 1 | 0 | 0 | 60.9 | 23.8 | 3 | | 33 | R | 3.4 | 3 | 2 | 1 | 1 | 1 | 1 | 0 | 0 | 31.1 | 4.2 | 2 | | 34 | R | 8.2 | 4 | n/a | 1 | 0 | 0 | 0 | 0 | 0 | 54.6 | 56.5 | 4 | | 35 | R | 1.2 | 3 | n/a | 1 | 0 | 0 | 0 | 1 | 0 | 26.0 | 7.8 | 2 | | 36 | R | 10.1 | 4 | n/a | 1 | 1 | 1 | 0 | 0 | 0 | 59.7 | 51.5 | 4 | | 37 | R | 5.3 | 3 | 4 | 1 | 1 | 1 | 0 | 0 | 0 | 28.6 | 41.1 | 3 | | 38 | R | 2.8 | 1 | n/a | 1 | 0 | 1 | n/a | [1] | 0 | 5.7 | 2.7 | 1 | | 39 | L | 6.8 | 4 | n/a | 1 | 0 | 1 | n/a | 1 | 0 | 10.0 | 27.2 | 3 | | 40 | L | 3.9 | 2 | 2 | 1 | 1 | 1 | n/a | 0 | 0 | 9.0 | 8.6 | 2 | | 41 | R | 12.5 | 2 | n/a | 1 | 0 | 1 | n/a | [1] | 0 | 4.3 | 4.1 | 2 | | 42 | L | 3.8 | 3 | 2 | 1 | 1 | 1 | 0 | 0 | 0 | 15.7 | 9.3 | 2 | | 43 | R | 5.8 | 2 | n/a | 1 | 0 | 1 | n/a | 0 | 0 | 5.7 | 5.3 | 2 | |
| --- | --- | --- | --- | --- | --- | --- | --- | --- | --- | --- | --- | --- | --- | --- | --- | --- | --- | --- | --- | --- | --- | --- | --- | --- | --- | --- | --- | --- | --- | --- | --- | --- | --- | --- | --- | --- | --- | --- | --- | --- | --- | --- | --- | --- | --- | --- | --- | --- | --- | --- | --- | --- | --- | --- | --- | --- | --- | --- | --- | --- | --- | --- | --- | --- | --- | --- | --- | --- | --- | --- | --- | --- | --- | --- | --- | --- | --- | --- | --- | --- | --- | --- | --- | --- | --- | --- | --- | --- | --- | --- | --- | --- | --- | --- | --- | --- | --- | --- | --- | --- | --- | --- | --- | --- | --- | --- | --- | --- | --- | --- | --- | --- | --- | --- | --- | --- | --- | --- | --- | --- | --- | --- | --- | --- | --- | --- | --- | --- | --- | --- | --- | --- | --- | --- | --- | --- | --- | --- | --- | --- | --- | --- | --- | --- | --- | --- | --- | --- | --- | --- | --- | --- | --- | --- | --- | --- | --- | --- | --- | --- | --- | --- | --- | --- | --- | --- | --- | --- | --- | --- | --- | --- | --- | --- | --- | --- | --- | --- | --- | --- | --- | --- | --- | --- | --- | --- | --- | --- | --- | --- | --- | --- | --- | --- | --- | --- | --- | --- | --- | --- | --- | --- | --- | --- | --- | --- | --- | --- | --- | --- | --- | --- | --- | --- | --- | --- | --- | --- | --- | --- | --- | --- | --- | --- | --- | --- | --- | --- | --- | --- | --- | --- | --- | --- | --- | --- | --- | --- | --- | --- | --- | --- | --- | --- | --- | --- | --- | --- | --- | --- | --- | --- | --- | --- | --- | --- | --- | --- | --- | --- | --- | --- | --- | --- | --- | --- | --- | --- | --- | --- | --- | --- | --- | --- | --- | --- | --- | --- | --- | --- | --- | --- | --- | --- | --- | --- | --- | --- | --- | --- | --- | --- | --- | --- | --- | --- | --- | --- | --- | --- | --- | --- | --- | --- | --- | --- | --- | --- | --- | --- | --- | --- | --- | --- | --- | --- | --- | --- | --- | --- | --- | --- | --- | --- | --- | --- | --- | --- | --- | --- | --- | --- | --- | --- | --- | --- | --- | --- | --- | --- | --- | --- | --- | --- | --- | --- | --- | --- | --- | --- | --- | --- | --- | --- | --- | --- | --- | --- | --- | --- | --- | --- | --- | --- | --- | --- | --- | --- | --- | --- | --- | --- | --- | --- | --- | --- | --- | --- | --- | --- | --- | --- | --- | --- | --- | --- | --- | --- | --- | --- | --- | --- | --- | --- | --- | --- | --- | --- | --- | --- | --- | --- | --- | --- | --- | --- | --- | --- | --- | --- | --- | --- | --- | --- | --- | --- | --- | --- | --- | --- | --- | --- | --- | --- | --- | --- | --- | --- | --- | --- | --- | --- | --- | --- | --- | --- | --- | --- | --- | --- | --- | --- | --- | --- | --- | --- | --- | --- | --- | --- | --- | --- | --- | --- | --- | --- | --- | --- | --- | --- | --- | --- | --- | --- | --- | --- | --- | --- | --- | --- | --- | --- | --- | --- | --- | --- | --- | --- | --- | --- | --- | --- | --- | --- | --- | --- | --- | --- | --- | --- | --- | --- | --- | --- | --- | --- | --- | --- | --- | --- | --- | --- | --- | --- | --- | --- | --- | --- | --- | --- | --- | --- | --- | --- | --- | --- | --- | --- | --- | --- | --- | --- | --- | --- | --- | --- | --- | --- | --- | --- | --- | --- | --- | --- | --- | --- | --- | --- | --- | --- | --- | --- | --- | --- | --- | --- | --- | --- | --- | --- | --- | --- | --- | --- | --- | --- | --- | --- | --- | --- | --- | --- | --- | --- | --- | --- | --- | --- | --- | --- | --- | --- | --- | --- | --- | --- | --- | --- | --- | --- | --- | --- | --- | --- | --- | --- | --- | --- | --- | --- | --- | --- | --- | --- | --- | --- | --- | --- | --- | --- | --- | --- | --- | --- | --- | --- | --- | --- | --- | --- | --- | --- | --- | --- | --- | --- | --- | --- | --- | --- | --- | --- | --- | --- | --- | --- | --- | --- | --- | --- | --- | --- | --- | --- |

^a^ Radiological and clinical SHEAR diagnoses were directly compared to the SHEAR measurements taken from the 3-D CT reconstructions. A ‘1’ was given for an accurate assessment within one grade of SHEAR, and ‘0’ was given for inaccuracy or lack of diagnosis. Actual SHEAR grades diagnosed by the radiologists or extrapolated from the reported percent affected scapula above the clavicle are noted. N/A indicates no grade of SHEAR was given.

^b^ Radiological reports were given a ‘1’ if there was a diagnosis of posterior subluxation, and a ‘0’ if it was not mentioned or misdiagnosed as ‘normal’. Only reports on patients who had more than a 10% difference in posterior subluxation between the affected and contralateral shoulders were included in this analysis. N/A indicates those patients with less than a 10% measured difference between the affected and contralateral shoulders.

^c^ Posterior subluxation was calculated as previously described  [14, 15]. The percentage reported here is the difference in posterior subluxation between the contralateral and affected shoulders.

^d^ From the anterior 3-D CT images, the percent of the total area of the scapula visible above the clavicle in the affected shoulder compared to the contralateral shoulder was measured as described previously  [4].
